# Association of Hepatic Steatosis Index with Nonalcoholic Fatty Liver Disease Diagnosed by Non-Enhanced CT in a Screening Population

**DOI:** 10.3390/diagnostics11122168

**Published:** 2021-11-23

**Authors:** Jieun Chung, Hee-Sun Park, Young-Jun Kim, Mi-Hye Yu, Sungeun Park, Sung-Il Jung

**Affiliations:** Department of Radiology, Konkuk University School of Medicine 120-1, Neungdong-ro, Gwangjin-gu, Seoul 05030, Korea; 20170073@kuh.ac.kr (J.C.); yjkim@kuh.ac.kr (Y.-J.K.); 20140130@kuh.ac.kr (M.-H.Y.); 20210126@kuh.ac.kr (S.P.); radsijung@kuh.ac.kr (S.-I.J.)

**Keywords:** hepatic steatosis, nonalcoholic fatty liver disease, CT, hepatic steatosis index, screening, metabolic syndrome

## Abstract

The noninvasive diagnosis of hepatic steatosis is of increasing concern. This study investigated the association of hepatic steatosis determined by non-enhanced CT criteria with clinical parameters in a screening population. Asymptomatic patients who underwent abdominal CT at our healthcare center were retrospectively analyzed (*n* = 339). Two radiologists measured the attenuation values of the liver parenchyma and spleen using non-enhanced CT images. CT criteria for hepatic steatosis were (a) absolute liver attenuation value <48 Hounsfield units (HU), (b) liver-to-spleen attenuation ratio <0.8, and (c) attenuation difference between the liver and spleen <−10. Body mass index (BMI) and hepatic steatosis index (HSI) were calculated, and laboratory findings were recorded. The association of hepatic steatosis with clinical parameters was assessed using univariate and logistic regression analyses. The presence of hepatic steatosis was significantly associated with the levels of serum fasting glucose and triglycerides, the alanine aminotransferase to aspartate aminotransferase (ALT/AST) ratio, BMI, and HSI values using any of the CT criteria. Logistic regression analysis revealed that the serum fasting glucose level and HSI were significantly associated with hepatic steatosis using criterion (a), while the ALT/AST ratio and HSI were associated with hepatic steatosis using criteria (b) and (c). The presence of hepatic steatosis on non-enhanced CT should be considered to indicate possible clinical profile abnormalities regarding metabolic syndrome.

## 1. Introduction

Nonalcoholic fatty liver disease (NAFLD) is defined as the accumulation of fat, mainly triglycerides, in hepatocytes without evidence of excessive alcohol consumption or other causes [1]. This includes a wide spectrum of fatty liver diseases, encompassing simple hepatic steatosis, steatohepatitis, fibrosis, and cirrhosis [2]. NAFLD is closely associated with insulin resistance, dyslipidemia, visceral obesity, and type 2 diabetes and is regarded as a hepatic manifestation of metabolic syndrome [3]. Since patients with NAFLD show increased risks of cardiovascular disease and liver-related mortality, early detection of NAFLD is essential to prevent progression to more advanced stages [4].

Various imaging modalities have been used for the assessment of hepatic steatosis. Ultrasonography provides fair accuracy for detecting moderate to severe hepatic steatosis but shows limited accuracy for detecting a mild degree of the disease [5]. In addition, the qualitative assessment and subjective nature of the examination bear the potential limitation of substantial intraobserver and interobserver variability [6]. Computed tomography (CT) facilitates the quantitative assessment of hepatic steatosis by measuring the liver attenuation value, expressed as Hounsfield units (HU) [7]. As the attenuation value of fat is much lower than that of soft tissue, the attenuation value of hepatic parenchyma decreases as hepatic steatosis develops and progresses [7]. CT scanning is preferred for abdominal screening owing to the limitations of ultrasound for the evaluation of deep-seated organs. Therefore, the diagnosis of hepatic steatosis using CT in health screening populations is increasing.

Several studies have investigated the association of NAFLD with clinical and biochemical parameters and devised prediction models [2,8,9,10,11,12]. One of the studies suggested the hepatic steatosis index (HSI) using liver enzyme levels and body mass index (BMI) for the screening of NAFLD in a certain screening population, which was based on hepatic steatosis diagnosed by ultrasonography [10]. The study revealed that HSI values showed a significant correlation with hepatic steatosis grade determined by ultrasonography. As mentioned above, the determination of hepatic steatosis by ultrasonography is not only subjective but also suffers from significant interobserver variability because the examination is performed by various radiologists.

Therefore, in this study, we aimed to investigate the association between hepatic steatosis determined by non-enhanced CT and clinical parameters, including HSI and laboratory parameters regarding metabolic syndrome, in a screening population.

## 2. Materials and Methods

### 2.1. Study Population and Data Collection

Between January 2019 and March 2019, a total of 394 asymptomatic adult patients underwent abdominal CT at the healthcare center of our institution for routine health screening. The routine health screening program included laboratory blood tests and body size measurements on the same day as the CT scanning. Among them, 55 patients were excluded from the study for the following reasons: (1) excessive alcohol consumption (*n* = 25; 20 g/day); (2) presence of malignancy (*n* = 12; breast cancer, colorectal cancer, pancreatic neuroendocrine tumor, lung cancer, melanoma, renal cell carcinoma, ovarian cancer, and thyroid cancer); (3) positive for serologic hepatitis B antigen (*n* = 4); (4) positive for serologic hepatitis C antibody (*n* = 1); (5) presence of active inflammation (*n* = 7; serum white blood cell count >10 × 10^3^/µL); (6) data for body size measurement was not available (*n* = 4); and (7) ongoing unconfirmed medication (*n* = 2).

**Figure 1:**
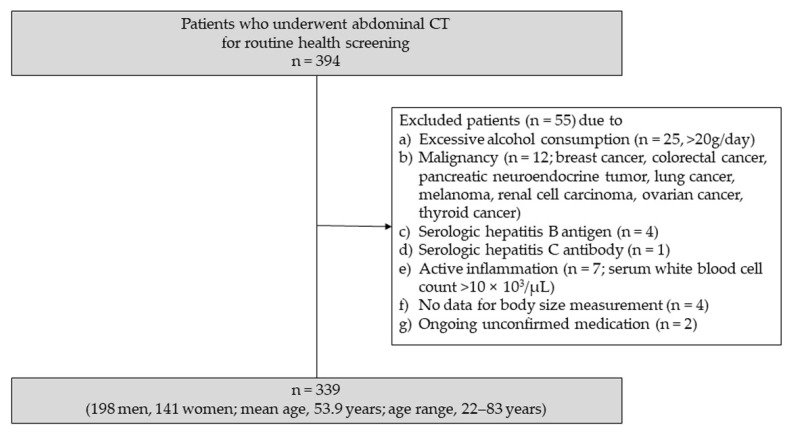
Flow chart of the patient population and study design.

Finally, 339 patients (198 men and 141 women; mean age, 53.9 years; age range, 22–83 years) who met these criteria were included in the study. Figure 1 displays a flow chart of the patient population and study design.

### 2.2. CT Examinations

The CT scans were performed using a 64-channel scanner (Ingenuity; Philips Healthcare, Cleveland, OH, USA). Non-enhanced images were obtained by employing beam collimation of 64 × 0.625 mm, a spiral pitch of 0.984, a tube voltage of 120 kVp, and a tube current of 200 mAs with an automatic exposure control (iDose; Philips Healthcare). Images were reconstructed at a section thickness of 3 mm and an interval of 3 mm.

### 2.3. Image Analysis

Two observers, including one of the faculty members from abdominal radiology (observer 1, 14 years of experience in abdominal radiology) and a third-year resident in the radiology department (observer 2) independently performed a quantitative analysis of non-enhanced CT images. The CT images were anonymized and randomized for review. Both readers were blinded to the results of the laboratory blood test and body size measurement.

**Figure 2:**
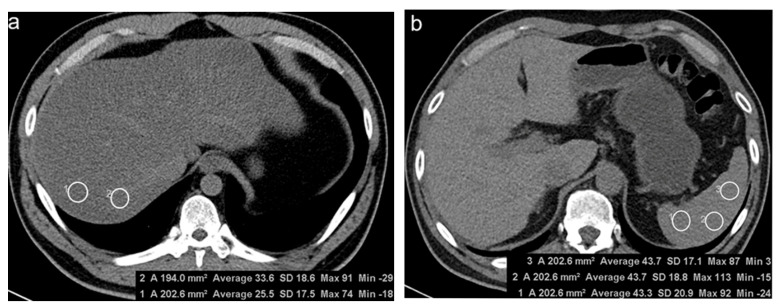
Measurement of hepatic (**a**) and splenic (**b**) attenuation on non-enhanced computed tomography. (**a**) Two 2 cm^2^ circular regions of interest (ROIs) were manually drawn at segment VII. (**b**) Three 2 cm^2^ ROIs were manually at three different sites in the middle third of the spleen.

Liver attenuation was obtained by averaging Hounsfield unit (HU) values of four 2 cm^2^ circular regions of interest (ROIs): two ROIs were manually drawn at two different sites in each of the posterior segments of the right hepatic lobe (hepatic segments VI and VII according to the Couinaud system) [13]. Splenic attenuation was measured by averaging HU values of three 2 cm^2^ circular ROIs placed at three different sites in the middle third of the spleen. To measure hepatic and splenic attenuation, ROIs were placed to exclude macroscopic hepatic vessels, biliary structures, and any focal liver lesions, and they were at least 5–10 mm away from the periphery of the liver [8] (Figure 2). The same measurement process was performed in two sessions, and each session was scheduled a week apart to minimize recall bias.

Three criteria using non-enhanced CT, which were proposed in the previous literature [5,8,14], were applied for the diagnosis of hepatic steatosis: (a) absolute liver attenuation value (HU_liver_) < 48, (b) the liver-to-spleen HU ratio (CT_L/S_) < 0.8, and (c) attenuation difference between the liver and spleen (CT_L-S_) < −10.

### 2.4. Clinical Information

Laboratory results for serum aspartate aminotransferase (AST), alanine aminotransferase (ALT), total triglycerides, total cholesterol, high-density lipoprotein (HDL), low-density lipoprotein (LDL), fasting glucose, glycated hemoglobin (HbA1c), and uric acid were documented. Body weight and height were measured, and BMI was calculated as weight (kg) divided by the squared value of height (m^2^). As the clinical index for predicting hepatic steatosis, the ALT/AST ratio was calculated by dividing serum ALT (IU/L) by AST (IU/L). HSI was calculated according to a previous study: HSI = 8 × ALT-AST ratio + BMI (+2 if diabetes; +2 if female) [10].

### 2.5. Statistical Analysis

Since the clinical and laboratory parameters expressed as continuous variables rejected the normal distribution (*p* < 0.001, Kolmogorov–Smirnov test), numerical data were presented as medians ± interquartile range (IQR), and CT values were presented as means ± standard deviation (SD). The values of three CT indices for hepatic steatosis were compared between the groups with and without hepatic steatosis using the Student’s *t*-test. The associations between the presence of hepatic steatosis using the three criteria mentioned in the Image Analysis subsection and each laboratory parameter, BMI, and HSI were performed using univariate analysis. On the basis of the results of the univariate analysis, a multiple logistic regression analysis was conducted to determine the association between these parameters and the presence of hepatic steatosis. Correlation analyses between HSI and each CT criteria were conducted. ROC curve analysis was performed to determine the optimal HSI level for the diagnosis of hepatic steatosis using the three CT criteria. To minimize the redundancy of the data presented, only the data from one observer (observer 1) were used in the analysis.

Intraobserver and interobserver variability were assessed using the intraclass correlation coefficient (ICC) to evaluate the measurement variability. An ICC of <0.20 indicated poor agreement; 0.20–0.40 indicated fair agreement; 0.40–0.60 indicated moderate agreement; 0.60–0.80 indicated good agreement; and >0.80 indicated excellent agreement. *p*-values of <0.05 were considered to indicate a statistically significant difference. Statistical analysis was performed using commercially available software (MedCalc^®^, version 19.5.3; MedCalc Software, Mariakerke, Belgium).

## 3. Results

### 3.1. Diagnosis of Hepatic Steatosis Using Three CT Criteria

When criterion (a) was applied, hepatic steatosis was present in 110 patients (32.4%, HU_liver_ 55.6 ± 4.3 (mean ± SD)) and was absent in 229 patients (67.6%, HU_liver_ 39.8 ± 8.9). Using criterion (b), hepatic steatosis was present in 34 patients (10.0%, CT_L/S_ 0.63 ± 0.20) and was absent in 305 patients (90.0%, CT_L/S_ 1.12 ± 0.15). Using criterion (c), 34 patients had hepatic steatosis (10.0%, CT_L-S_ −17.48 ± 7.82), while 305 patients did not have hepatic steatosis (90.0%, CT_L-S_ 5.34 ± 4.70). The values of the CT indices were significantly different between the groups with and without hepatic steatosis (*p* < 0.001).

### 3.2. Association of Clinical and Laboratory Parameters with Hepatic Steatosis

The characteristics of the study populations with and without hepatic steatosis using the three criteria are described in detail (Table 1, Table 2 and Table 3). Using criterion (a), the levels of AST, ALT, fasting glucose, HDL, triglycerides, and uric acid, as well as the values of the ALT/AST ratio, BMI, and HSI, were significantly different between the two groups. Using criterion (b), levels of ALT, fasting glucose, HDL, triglycerides, total cholesterol, and uric acid, as well as the values of the ALT/AST ratio, BMI, and HSI, were significantly different between the groups. Using criterion (c), the levels of fasting glucose, Hb1Ac, triglycerides, total cholesterol, and uric acid, as well as the ALT/AST ratio, BMI, and HSI values, were significantly different between the two groups.

When the logistic regression analysis was performed using the significant parameters, the fasting glucose level and HSI were significantly associated with hepatic steatosis using criterion (a), while the ALT/AST ratio and HSI were significantly associated with hepatic steatosis using criteria (b) and (c).

### 3.3. Correlation between HSI and the Three CT Criteria

**Figure 3:**
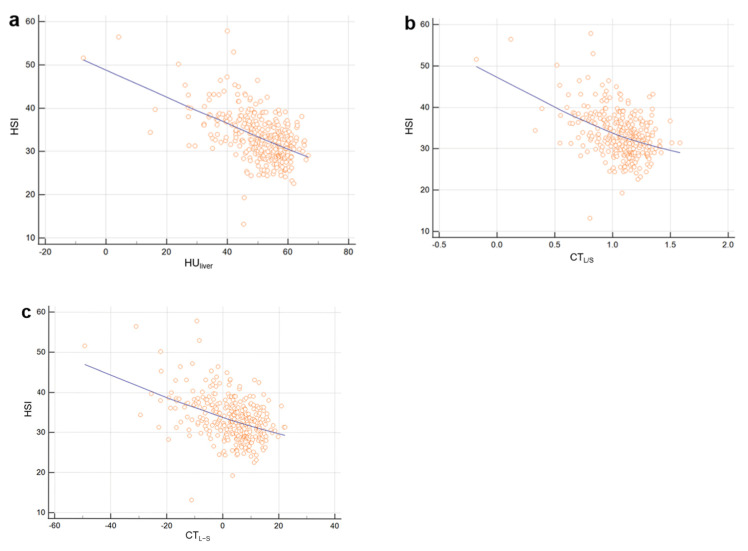
Scatter plots of HSI level as a function of HU_liver_ (**a**), CT_L/S_ (**b**), and CT_L-S_ (**c**). Solid lines represent best-fit linear regression. HSI showed moderate negative correlation with criterion (**a**) (Spearman’s coefficient of rank correlation rho (*r*) = −0.531) and showed weak negative correlations with criteria (**b**) and (**c**) (*r* = −0.436 and −0.437, respectively).

HSI showed a moderate negative correlation with criterion (a) (Spearman’s coefficient of rank correlation rho (*r*) = −0.531), and the correlation was statistically significant (*p* < 0.0001). HSI showed weak negative correlations with criteria (b) and (c) (*r* = −0.436 and −0.437, respectively), with statistically significant correlation (*p* < 0.001) (Figure 3).

### 3.4. Diagnostic Performance of HSI for Hepatic Steatosis Using the Three CT Criteria

**Figure 4:**
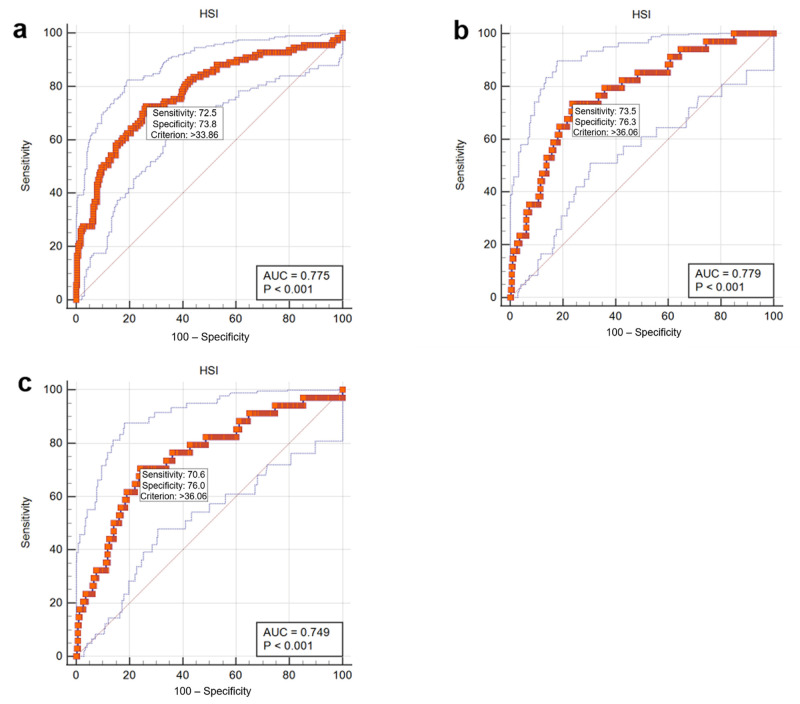
Receiver operating characteristics curves showing the performance of HSI in the diagnosis of hepatic steatosis using CT criteria (**a**–**c**).

The area under the ROC curve (A*_z_*) of HSI for the diagnosis of hepatic steatosis was 0.775 (95% confidence interval (CI), 0.728–0.820) with a sensitivity 72.5% and a specificity 73.8% at a cutoff value of 33.86 (*p* < 0.001) using criterion (a); 0.779 (95% confidence interval (CI), 0.730–0.822) with a sensitivity 73.5% and a specificity 76.3% at a cutoff value of 36.06 (*p* < 0.001) using criterion (b); and 0.749 (95% confidence interval (CI), 0.699–0.794) with a sensitivity 70.6% and a specificity 76.0% at a cutoff value of 36.06 (*p* < 0.001) using criterion (c) (Figure 4).

### 3.5. Intraobserver and Interobserver Agreement

The intraobserver agreement for the measurement of liver and spleen attenuation was good for observer 1 (ICCs of 0.71 and 0.64, respectively) and excellent for observer 2 (ICCs 0.95 and 0.95, respectively). The interobserver agreement of the liver and spleen measurements ranged from good to excellent between the two observers (ICCs of 0.96 and 0.76, respectively).

## 4. Discussion

Our study results demonstrated that hepatic steatosis using the three non-enhanced CT criteria showed significant associations with various laboratory parameters and clinical indices regarding metabolic syndrome, including the serum fasting glucose level, triglyceride level, ALT/AST ratio, BMI, and HSI. Previous studies have reported that hepatic steatosis on CT is associated with dyslipidemia, the presence of diabetes, alcoholism, and hypertension [15,16]. Other recent studies have shown that incidentally detected hepatic steatosis determined by various CT criteria is associated with an abnormal lipid profile, abnormal liver function, and certain comorbidities [8,9]. In our study, we sought to validate the association in a health screening population, specifically in the setting of NAFLD, by excluding those with hepatitis B or C virus infection or alcoholism. We found that associated clinical and laboratory parameters were mainly related to metabolic syndrome, which is also known as insulin resistance syndrome. Among these parameters, HSI is a recently devised clinical index for the screening of NAFLD [10]. It includes the ALT/AST ratio, BMI, the presence of diabetes, and female sex. The ALT/AST ratio is known as a marker for insulin resistance, while the AST/ALT ratio is more often elevated in alcoholic liver disease [10,17]. Using multiple logistic regression analysis, our study revealed that HSI was significantly associated with the presence of hepatic steatosis when all three criteria were applied. In addition, the ALT/AST ratio was found to be significantly associated with hepatic steatosis diagnosed using CT_L/S_ and CT_L-S_ criteria.

The diagnosis of hepatic steatosis using CT has been reported in several previous studies. There are four criteria using non-enhanced CT, including HU_liver_, CT_L/S_, CT_L-S_, and higher hepatic vessel attenuation than liver parenchyma attenuation [8,15,18,19]. When using enhanced CT, hepatic steatosis is defined as an attenuation difference between the liver and spleen of <−20 in the portal venous phase [8,20]. A literature search revealed that HU_liver_ measurement on non-enhanced CT best predicted pathologic liver fat content and showed a stronger correlation with the histologic degree of hepatic steatosis CT_L-S_ in some studies [19,21,22]. However, HU_liver_ may be subject to errors in attenuation variability across different CT vendors, and this error can be avoided through CT_L/S_ or CT_L-S_ methods using spleen attenuation as an internal calibration [19,23]. A few studies have reported that portal phase images of contrast-enhanced CT showed comparable accuracy in the diagnosis of hepatic steatosis; however, it has potential errors regarding liver attenuation variability related to contrast injection methods and scan timing [5,24]. Therefore, non-enhanced CT is usually preferred, and the two most frequently used methods are HU_liver_ and CT_L-S_ [23].

When our study adopted the threshold value based on the reference of a previous study (HU_liver_ = 48, CT_L/S_ = 0.8, and CT_L-S_ = −10, the proportion of hepatic steatosis was much higher with criterion (a) (32.4%) than with criteria (b) and (c) (10.0% in both). The reason for the difference in incidence between the groups can be partly explained by the fact that the three indices are not based on histologic reference [8,9]. Considering the previous study for hepatic steatosis using non-enhanced CT based on histologic results as a reference standard, CT indices based on both liver and spleen attenuation showed higher diagnostic performance than the one based on liver attenuation alone [25]. A threshold value of −10 for CT_L-S_ is somewhat rigorous compared with other studies [23,26], and variability of threshold values for CT criteria exists depending on the methods and study populations [5,23,26,27]. One study reported that an HU_liver_ of 48 and a CT_L-S_ of −2 were threshold values of moderate to severe steatosis with a specificity of 100% [23], while another study reported CT_L-S_ of −9 with 100% specificity and 82% sensitivity [27]. Some studies even applied threshold values of >0 [5,26]. The CT criteria used in our study were designed to be more specific to moderate to severe hepatic steatosis according to previous studies [8,9,23], especially CT_L/S_ and CT_L-S_. Further investigation that adopts different cutoff values for CT_L/S_ and CT_L-S_ based on other reference literature is expected to show more relevant results.

In our study, the cutoff levels of HSI for the prediction of hepatic steatosis were 36.06 using the criteria (b) and (c). Interestingly, this result is in accord with a previous study on the validation of HSI [10]. According to the study, the subject was unlikely to have NAFLD if the HSI was <30.0, while the subject could be considered to have NAFLD if the HSI was >36.0. Since HSI validation was based on US imaging, which is considered to be an imperfect gold standard for the diagnosis of hepatic steatosis because of subjectivity, we sought to verify the result using more objective criteria. HSI level measurement was reproducible using the CT criteria in our study, and this suggests that CT indices can be applied to predict moderate to severe hepatic steatosis as well as clinical parameters indicating metabolic syndrome. However, in contrast to the previous study [10], AUCs of HSI are quite low in our study, with poor sensitivity and specificity, and this result makes HSI not reliable for the diagnosis of NAFLD in health screening population. The contradictory results may be attributed to many factors, such as different reference imaging modality, different method of determining hepatic steatosis, or lack of histologic result of the hepatic steatosis.

Our study has several limitations. First, this was a retrospective study with a lack of pathological confirmation of hepatic steatosis, which is the gold standard for this disease. However, the study was designed under the setting of a health screening population; therefore, pathologic data were neither available nor relevant to real clinical practice. Second, the degree of hepatic steatosis (mild, moderate, and severe) was not stratified in the study. One potential limitation of CT is its low accuracy in detecting a mild degree of steatosis, and this method may not be suitable for the evaluation of NAFLD because patients with NAFLD frequently have a mild degree of hepatic steatosis [28,29]. The CT index criteria used in our study correspond to the diagnosis of moderate to severe hepatic steatosis, which is a major health concern. In this regard, our study seems to be meaningful, as it validated the significant association between the group with hepatic steatosis and clinical and laboratory parameters indicating metabolic syndrome. Third, the potential hazard of radiation makes CT unsuitable for monitoring patients with NAFLD [23]. However, there is an increase in the use of CT scanning instead of ultrasonography in the health screening population owing to the limitations of ultrasound for the evaluation of deep-seated organs, such as the pancreas, which is of substantial concern nowadays. Therefore, the evaluation of hepatic steatosis using CT is available without additional radiation exposure in these populations. In addition, nonenhanced magnetic resonance imaging protocol optimized for hepatic steatosis screening, such as chemical shift sequence, proton density fat fraction, or magnetic resonance spectroscopy should be considered as a substitute for CT, which is free from radiation issue.

## 5. Conclusions

In conclusion, the presence of hepatic steatosis, especially of a moderate to severe degree, diagnosed by non-enhanced CT criteria in a screening population should be considered to have a relationship with clinical and laboratory profile abnormalities, including HSI, which indicate metabolic syndrome. Vice versa, CT measurement of fat in the liver may be useful for patients at risk of metabolic syndrome.

## Figures and Tables

**Table 1:** Comparison of clinical parameters between the two groups determined by the hepatic steatosis criterion (a; HU_liver_ < 48).

	Patients with Hepatic Steatosis (*n* = 110)	Patients without Hepatic Steatosis (*n* = 229)	*p*-Value	
			Univariate Analysis	Multivariate Analysis
AST (IU/L)	29 ± 15	25 ± 11	0.041	0.143
ALT (IU/L)	33 ± 31	24 ± 12	<0.001	0.22
Fasting glucose (mg/dL)	104.5 ± 27	97 ±16	<0.001	0.008
HbA1c (%)	5.9 ± 1.18	5.6 ± 0.4	0.071	
Triglyceride (mg/dL)	133 ± 103.25	87 ± 66	<0.001	0.366
Total cholesterol (mg/dL)	205 ± 76.5	203 ± 53	0.172	
HDL (mg/dL)	51 ± 16.5	59 ± 21	<0.001	0.326
LDL (mg/dL)	128 ± 64.5	121 ± 47	0.514	
Uric acid (mg/dL)	5.5 ± 1.63	4.7 ± 1.6	0.001	0.415
ALT/AST ratio	1.18 ± 0.53	0.9 ± 0.33	<0.001	0.235
HSI	36.9 ± 6.69	31.4 ± 5.1	<0.001	0.038
BMI (kg/m^2^)	25.85 ± 4.2	23.2 ± 3.9	<0.001	0.698

Data are medians ± IQR values. AST: aspartate aminotransferase, ALT: alanine aminotransferase, HbA1c: hemoglobin A1c, HDL: high-density lipoprotein, LDL: low-density lipoprotein, HSI: hepatic steatosis index, BMI: body mass index, IQR: interquartile range.

**Table 2:** Comparison of clinical parameters between the two groups determined by the hepatic steatosis criterion (b; CT_L/S_ < 0.8).

	Patients with Hepatic Steatosis (*n* = 34)	Patients without Hepatic Steatosis (*n* = 305)	*p*-Value	
			Univariate Analysis	Multivariate Analysis
AST (IU/L)	31.5 ± 16.5	25 ± 12	0.158	
ALT (IU/L)	40 ± 35	25 ± 14	0.043	0.571
Fasting glucose (mg/dL)	111.5 ± 36.5	98 ± 18	<0.001	0.134
HbA1c (%)	6.25 ± 1.25	5.6 ± 0.5	0.82	
Triglyceride (mg/dL)	152 ± 146.5	95 ± 81	<0.001	0.311
Total cholesterol (mg/dL)	207.5 ± 78	203 ± 55	0.005	0.436
HDL (mg/dL)	46 ± 17.75	57 ± 21	0.001	0.361
LDL (mg/dL)	120.5 ± 65.25	123 ± 50	0.754	
Uric acid (mg/dL)	5.35 ± 2	4.8 ± 1.85	0.02	0.214
ALT/AST ratio	1.3 ± 0.53	0.94 ± 0.36	0.003	0.037
HSI	38.02 ± 5.51	32.54 ± 6.78	<0.001	0.037
BMI (kg/m^2^)	26 ± 3.93	23.6 ± 4.4	<0.001	0.102

Data are median ± IQR values. AST: aspartate aminotransferase, ALT: alanine aminotransferase, HbA1c: hemoglobin A1c, HDL: high-density lipoprotein, LDL: low-density lipoprotein, HSI: hepatic steatosis index, BMI: body mass index, IQR: interquartile range.

**Table 3:** Comparison of clinical parameters between the two groups determined by the hepatic steatosis criterion (c; CT_L-S_ < −10).

	Patients with Hepatic Steatosis (*n* = 34)	Patients without Hepatic Steatosis (*n* = 305)	*p*-Value	
			Univariate Analysis	Multivariate Analysis
AST (IU/L)	32 ± 16.5	25 ± 12	0.151	
ALT (IU/L)	38.5 ± 35.5	25 ± 14	0.053	
Fasting glucose (mg/dL)	110.5 ± 29.25	98 ± 18	0.004	0.292
HbA1c (%)	6.25 ± 1.25	5.6 ± 0.5	0.002	0.891
Triglyceride (mg/dL)	160 ± 148.25	94 ± 83	<0.001	0.11
Total cholesterol (mg/dL)	207.5 ± 78	203 ± 55	0.006	0.391
HDL (mg/dL)	46 ± 19	57 ± 20	0.052	
LDL (mg/dL)	120.5 ± 65.25	123 ± 50	0.957	
Uric acid (mg/dL)	5.4 ± 2	4.8 ± 1.9	0.005	0.111
ALT/AST ratio	1.3 ± 0.53	0.94 ± 0.37	0.006	0.028
HSI	37.68 ± 6.26	32.54 ± 6.78	<0.001	0.028
BMI (kg/m^2^)	26 ± 4.18	23.6 ± 4.3	0.001	0.081

Data are median ± IQR values. AST: aspartate aminotransferase, ALT: alanine aminotransferase, HbA1c: hemoglobin A1c, HDL: high-density lipoprotein, LDL: low-density lipoprotein, HSI: hepatic steatosis index, BMI: body mass index, IQR: interquartile range.

## Data Availability

Not available.
